# ICD-10-Coding of Medically Unexplained Physical Symptoms and Somatoform Disorders—A Survey With German GPs

**DOI:** 10.3389/fmed.2021.598810

**Published:** 2021-03-30

**Authors:** Nadine J. Pohontsch, Thomas Zimmermann, Marco Lehmann, Lisa Rustige, Katinka Kurz, Bernd Löwe, Martin Scherer

**Affiliations:** ^1^Department of General Practice and Primary Care, University Medical Center Hamburg-Eppendorf, Hamburg, Germany; ^2^Department of Psychosomatic Medicine and Psychotherapy, University Medical Center Hamburg-Eppendorf, Hamburg, Germany; ^3^Institute for Sex Research, Sexual Medicine and Forensic Psychiatry, University Medical Center Hamburg-Eppendorf, Hamburg, Germany; ^4^Department of Cardiology, University Heart and Vascular Center Hamburg, Hamburg, Germany

**Keywords:** general practice, primary care (MeSH), somatoform disorder, diagnosis, coding (ICD), survey

## Abstract

**Background:** General practitioners (GPs) are reluctant to use codes that correspond to somatization syndromes.

**Aim:** To quantify GPs' views on coding of medically unexplained physical symptoms (MUPS), somatoform disorders, and associated factors.

**Design and Setting:** Survey with German GPs.

**Methods:** We developed six survey items [response options “does not apply at all (1)”—“does fully apply (6)”], invited a random sample of 12.004 GPs to participate in the self-administered cross-sectional survey and analysed data using descriptive statistics and logistic regression analyses.

**Results:** Response rate was 15.2% with *N* = 1,731 valid responses (54.3% female). Participants considered themselves familiar with ICD-10 criteria for somatoform disorders (*M* = 4.52; *SD* =.036) and considered adequate coding as essential prerequisite for treatment (*M* = 5.02; *SD* = 1.21). All other item means were close to the scale mean: preference for symptom or functional codes (*M* = 3.40; *SD* = 1.21), consideration of the possibility of stigmatisation (*M* = 3.30; *SD* = 1.35) and other disadvantages (*M* = 3.28; *SD* = 1.30) and coding only if psychotherapy is intended (*M* = 3.39; *SD* = 1.46). Exposure, guideline knowledge, and experience were most strongly associated with GPs' self-reported coding behaviour.

**Conclusions:** Subjective exposure, guideline knowledge, and experience as a GP, but no sociodemographic variable being associated with GPs' subjective coding behaviour could indicate that GPs offer a relatively homogeneous approach to coding and handling of MUPS and somatoform disorders. Strengthening guideline knowledge and implementation, and practise with simulated patients could increase the subjective competence to cope with the challenge that patients with MUPS and somatoform disorders present.

## Introduction

In Germany, the life-time-prevalence of somatoform disorders or syndromes is around 16% (1). There is considerable comorbidity with mental disorders such as anxiety or depression (2), but this does not explain enough variance to justify consideration as a “common mental disorder” (3). Recognising and labelling medically unexplained symptoms (MUPS) can be difficult for GPs (4) and prevalence rates of MUPS differ according to concepts and criteria used (5). For primary care, a meta-analysis (6) reported point prevalence rates of 26.2% for patients with at least one somatoform disorder according to DSM or ICD and 12-months prevalence of 18.9%. Lifetime prevalence was estimated at 41%. For at least one MUPS, the point prevalence was 40.2% and 12-months prevalence was estimated at 49%. Looking at persistent MUPS, Verhaak et al. (7) found a prevalence of ~2% (≥4 presentations of MUPS/year). Aamland et al. (8) found a 3% consultation prevalence rate for MUPS with >3months duration and function loss. All in all, we can conclude that MUPS and somatoform disorders are common in general practice.

In primary care medicine, mental disorders are often detected and treated without being explicitly labelled as or translated to coded diagnoses (9–12). There is general agreement, that valid and integer coding is a prerequisite for efficient, safe, and sound patient care (13–17), and research purposes such as the design and evaluation of health care interventions based on morbidity data (18, 19). Either way, we know that general practitioners (GP) often recognise their patients' MUPS (5) and care for patients' suffering (9). GPs describe uncertainty (about diagnoses) as a relevant aspect of their daily business and have reasons to not always code MUPS or a somatoform condition or disorder when facing certain diagnostic clues (9, 20).

Eisenberg sees diagnosis and treatment as a social act influenced by a physicians' character(istics) and patient variables (21). The attitudes of primary care providers towards mental disorders, the way they deal with them and their subjective competencies may differ according to gender, age, training years, years of experience, location of the practice (22–26) and beliefs about the disorder and subjective ability to offer help or treatment (27). Treatment decisions in chronic pain care appear to depend on the experience of providers (28) while preferences for diagnoses (29) depend mainly on physicians' specialty.

In the German health care system it is mandatory to record a patient's symptom(s) or disease(s) after a consultation using a four-digit-code from the ICD-10 (30). Not every noted ICD-10-code indicates a diagnosis as many ICD-10-codes label symptoms and codes indicating a diagnosis can be described as being tentative. Therefore coding behaviour may not to be equated to full extent with approaches to diagnosing. A qualitative study conducted by our team (9) showed that GPs have certain ways to handle coding of MUPS and somatoform disorders. We found GPs to be challenged by the process of coding MUPS and somatoform disorders. The GPs in our qualitative study described that coding is done for reimbursement reasons and that documented codes do not necessarily correspond fully to a patient's electronic health record. They code certain diagnoses to obtain reimbursement for more time-consuming consultations or when they plan to refer patients to psychotherapy (making at least a tentative diagnosis necessary). GPs are reluctant to code certain diagnoses when they fear stigma and other negative consequences for their patients (e.g., concerning life/health insurance contracts or a career as a civil servant) and seem to prefer suspect/tentative diagnoses and symptom coding to confirmed diagnoses (9). Insufficient knowledge of ICD-10-criteria, time pressure, or the use of heuristics may also lead to inadequate or inaccurate coding. GPs seem to accept diagnostic uncertainty as an elementary part of their work and consider ICD-10-coding as a not always necessary for treatment (9). These findings give insights about German GPs coding behaviour in the field of MUPS and somatoform disorders and may explain some of the commonly seen differences between ICD-10-driven routine and epidemiological data (31, 32).

It is important to know how GPs deal with the coding of MUPS and somatoform disorders and what influences coding behaviour. The aim of the study was therefore to evaluate the GPs' (dis-)agreement to different aspects of coding of MUPS and somatoform disorders and to identify associated variables by surveying German GPs.

## Materials and Methods

### Study Design

This survey was part of the project “Identification of barriers and difficulties involved in the process of diagnosing somatic symptom disorders in primary care” (BeSSD-GP). This mixed-methods project (33) consists of focus groups with GPs (9), interviews with patients and GPs, and a survey of GPs. This paper reports the results of the quantitative part of a sequential exploratory mixed methods design [Instrument Development Model, (34)] following the STROBE statement (35). Since qualitative data are suitable for identifying and describing phenomena (36), but not for describing how prominent they are and what influences them, we decided to follow up the qualitative data collection (9) with a survey of GPs. The German Research Foundation funded the study (http://gepris.dfg.de/gepris/projekt/276028312) and we registered the survey at the German Clinical Trials Register (DRKS00012942).

### Ethical Approval

We received ethical approval from the Ethics Committee of the Hamburg Medical Association (Germany, PV4763).

### Sampling, Recruitment and Data Collection

In Germany independent GPs (not associated to clinics or hospitals) provide primary care for the general population. We aimed at surveying all GPs registered in Germany. We contacted all 17 Associations of Statutory Health Insurance Physicians (ASHIP) in order to gather contact information from the GPs registered there. We received contact information from eight ASHIP therefore the sampling frame consisted of *N* = 15.389 GPs (out of 54.741 registered in the year 2017) and included all active GPs from eight (out of 17) statutory health care regions in Germany. Besides the contact information we received no further information about the GPs.

The GPs were randomly sampled (stratified proportionally for statutory health region of GPs' registration, *n* = 12.004) and invited to participate. GPs were eligible to participate if they worked as primary care physicians, we did not apply any other eligibility criteria, e.g., interest in somatoform disorders or association to a university. Participation was voluntary and anonymous. We offered no incentives.

The survey questionnaire was web-based and available in printed form. Following the Cochrane recommendations (37) to increase response rate, all eligible GPs were contacted by mail three times between 02/2018 and 05/2018. GPs received a primer postcard announcing that they would receive a survey questionnaire a week later and provided an URL leading to the web-based questionnaire. GPs then received the study package by mail, including an information sheet (study information, data protection and voluntariness of participation), the URL (we provided the same study information there), a printed questionnaire, and a stamped envelope. Two weeks later, all GPs received the entire package a second time. GPs willing to participate in the study were asked to either fill out the web-based questionnaire or return the filled in printed questionnaire using the return envelope.

### Survey Questionnaire

In the qualitative study described in detail elsewhere (9) we conducted six focus groups with German GPs to explore their views on coding and reasons for not coding MUPS and somatoform disorders. GPs were questioned using a semi-structured topic guide and the data was analysed using structuring content analysis with deductive and inductive coding. Three main topics were identified to be relevant: benefits of coding, restrained coding and coding inaccurately [(9), see also paragraph four of the section Introduction].

In order to be able to quantify GPs views on coding of MUPS and somatoform disorders, we used the results of our qualitative study (9), to develop six survey items (see Table 1). Item response options ranged from “does not apply at all” (1) to “does fully apply” (6). The questionnaire contained further questions on the topic of handling MUPS and somatoform disorders (not reported here) as well as questions on respondents' and practice attributes.

**Table 1:** Verbatim of coding Items.

**Item No**.	**Wording**
C1	I do not consider recording an ICD-10 code from chapter F 45.-(somatoform disorders) necessary to treat a patient with a somatoform disorder adequately.
C2	I am not familiar with the diagnostic criteria for a somatoform disorder according to the ICD-10.
C3	I rather record an ICD-10 code from chapter F 45.- (somatoform disorders) when I intend to refer a patient to psychotherapy.
C4	I prefer diagnoses that I perceive as less stigmatising for my patients.
C5	For patients with persistent, non-specific and bothersome somatic symptoms, I prefer to code symptoms and functional disorders instead of confirmed diagnoses.
C6	I prefer diagnoses that I consider as less compromising for the patients' further life course (for example, insurances, civil service career).

Items were developed by ML, NP, LR, and TZ, reviewed by all authors (psychologists/medical doctors) and in cognitive interviews (38) with three GPs (two female, one male; with varying experience working as a GP). There were no changes due to cognitive pretesting. Two hundred GPs received the questionnaire for quantitative pretesting. The evaluation of 35 returned questionnaires did not result in any changes, as there were no conspicuous patterns of missing values or comments indicating problems.

### Data Analysis

Descriptive statistics were used to describe the respondents' characteristics and survey items [scaled from 1 (does not apply) to 6 (does fully apply; mean 3.5)]. Two items (C1, C2) had to be inverted. We calculated the mean values of all six coding items with 95%-confidence intervals and examined distribution and skewness. To assess the chances of agreement or disagreement, we dichotomized the responses of the items in disagreement (values 1–3), and agreement (values 4–6). The categories of coding items were then analysed in logistic regressions, using gender, years of experience as a GP, GP-certified professional training, medical education in basic psychosomatic care, knowledge of guidelines, practice setting, subjective assessment of the proportion of patients with somatoform disorders ≥10% as covariates. Tests of interaction of the covariates revealed a relevant interaction of the covariates gender and experience as a GP. We included the interaction term in our regression models. Missing values are documented in Table 2. We assume that the missing values occured completely at random. We used Stata 16 for analyses.

**Table 2:** Sample characteristics (*N* = 1,731, categorical variables).

**Sex**	***N***	**%**
Female	939	54.3
Male	759	43.8
missing	33	1.9
**Proportion of GPs, certified by a 5-year advanced vocational training**	***N***	**%**
No	549	31.7
Yes	1,074	62.1
Missing	108	6.2
Medical education in basic psychosomatic care^*^	***N***	**%**
Yes	1,238	71.5
No	181	10.5
Missing	312	18.0
Knowledge of guidelines^**^	***N***	**%**
Rather yes	621	35.8%
Rather no	1,110	63.3%
Missing	15	0.9
**Practice setting**	***N***	**%**
Single practice	841	48.6
Group practice (separate accounting)	153	8.8
Group practice (joint accounting)	595	34.4
Medical care centre	65	3.8
Other	29	1.7
Missing	48	2.8
**Subjective rating of proportion of patients with somatoform disorders**	***N***	**%**
≤10%	718	41.5
>10%	682	39.4
Missing	331	19.1

* *This describes an additional qualification to identify and treat psychological and psychosomatic disorders for German general practitioners. It is acquired by completing a 50 h-seminar plus 30 h of balint group meetings*.

** *I know the recommendations of the AWMF-Guideline “Non-specific, functional and somatoform bodily complaints.” [(39), “does not apply at all” (1) to “does fully apply” (6)]*.

## Results

### Participants and Sample

From our sample of 12,004 PCPs, we received responses from 1,829 PCPs (15.2%). Reasons given for non-participation included retirement, death of the GP and subjective inappropriateness of the survey. Of the 1,829 responders, we omitted 98 due to missing responses to any of the coding items. Finally, 1,731 data sets were included. GPs had an average professional experience of 18.23 years (*SD* = 10.6). Further sample characteristics are shown in Table 2.

### Main Results

The proportions of agreement and disagreement (dichotomized values) with the six coding items are shown in Table 3. Mean values, proportions of original values and distributions are shown in Supplementary Figure 1.

**Table 3:** Proportions of agreement to coding Items.

**Item**	**Dichotomized value**	***N***	**%**
C1	Disagree	181	10.5
	Agree	1,537	89.5
C2	Disagree	413	24.0
	Agree	1,309	76.0
C3	Disagree	879	51.1
	Agree	842	48.9
C4	Disagree	935	54.4
	Agree	783	45.6
C5	Disagree	885	51.5
	Agree	832	48.5
C6	Disagree	957	55.6
	Agree	765	44.4

Four items (C3, C4, C5, and C6) remain slightly below the mean scale value (3.5) whereas two items (C1, C2) are negatively skewed. For items C3, C4, C5, and C6, the mean value of the items is close to the mean value of the scale, and agreement and disagreement with these items are almost equally distributed in the population. The Supplementary Table 1 shows the results of the regression analyses.

#### (C1) Diagnosis Necessary for Treatment

C1, with a mean value of 5.02, was heavily skewed in the direction of agreement. Odds for agreement were increased by 72% (*p* = 0.013) when GPs reported that more than 10% of their patients were perceived as having a somatoform disorder.

#### (C2) Familiar With Diagnostic Criteria

C2 was skewed in the direction of agreement, with a mean of 4.52. Knowledge of guidelines increased odds for agreement by 18% (*p* < 0.001). When GPs reported that more than 10% of their consulting patients were perceived as having a somatoform disorder odds of agreement increased by 41% (*p* = 0.022).

#### (C3) Coding for Therapy Only

C3 was distributed almost equally between disagreement and agreement, with the mean value of items (3.39) being close to the scale mean (3.5). None of the covariates tested showed a significant influence on this variable.

#### (C4) Prefer Less Stigmatising Diagnosis

C4 (mean value 3.30), was almost equally distributed between disagreement and agreement. Every 10 years of experience as a GP increased agreement to preferring non-stigmatising diagnoses by 20% (*p* = 0.049).

#### (C5) Prefer Coding Functional

C5 was evenly distributed between disagreement and agreement (mean 3.40). Knowledge of guidelines showed a significant influence on the preference of symptom and functional disorder codes instead of diagnoses. The odds for agreement were reduced by 7% (*p* = 0.034) for each unit increase in subjective knowledge of the guidelines.

#### (C6) Creating Less Sensitive Data

C6 was distributed almost equally between disagreement and agreement (mean 3.28). None of the covariates showed a significant influence on this item.

## Discussion

### Summary

German GPs were asked to report on their coding behaviour concerning MUPS and somatoform disorders. We received 1,731 valid answers. In general, the responding GPs agree with being familiar with the diagnostic criteria of somatoform disorders (C2). Guideline knowledge and higher subjective exposure (represented by perceived share of patients with somatoform disorders in practice) increased reports of familiarity. Coding specific diagnoses is generally considered necessary for adequate treatment (C1) and agreement increases with higher exposure. Almost equal proportions of GPs agree and disagree to use codes for somatoform disorders not only if they intend to refer patients to further (psycho-) therapy (C3, not influenced by any tested covariates) and the same is true for being influenced by the compromising (C6, not influenced by any tested covariates) and stigmatising potential (C4, odds for agreement increasing with experience) of these codes. Again, almost equal shares of surveyed GPs report rather not preferring symptom and functional disorder codes to confirmed diagnoses (C5, odds for agreement decreasing with increased guideline knowledge). All in all, in our study exposure, knowledge of guidelines, and experience as a GP were most strongly associated with the self-reported coding behaviour and preferences of GP's.

### Strengths and Limitations

This is the first survey conducted to quantify GPs' subjective coding behaviour regarding MUPS and somatoform disorders. We have created the items based on a comprehensive qualitative study and pretested them to ensure comprehensibility. The strength of the survey was to allow GPs from all over Germany to participate, thus creating a comprehensive nationwide image of GPs' views. GPs' reporting on their coding behaviour may have been influenced by social desirability bias (40), but we believe that the anonymous administration and processing of the questionnaire and non-judgmental wording of the items may have reduced such tendencies (40).

Despite all evidence-based efforts (37), the response rate in our study was rather low (15.2%). Since participation was voluntary, it cannot be ruled out that a self-selection bias may have led to GPs more interested in MUPS and somatoform disorders being over-represented in our sample. Unfortunately we were not able gather any information on that and it would be hard to tell how the data was influenced by that. Either way, the sample is comparable in terms of gender and medical specialty to the population of GPs in Germany (41) [data not shown].

### Comparisons With Existing Literature

Compared to the qualitative data (9), our survey data show a slightly different picture of the coding behaviour of GPs. This could be due to the focus on problematic areas and behaviours in the focus groups (9), which could reinforce the impression that massive problems exist in certain areas. This makes it all the more important to quantify the phenomena identified in qualitative studies to be able to adequately assess their significance (34). While our focus group guide (9) included patient vignettes and the discussion often focused on certain anonymous patients, the way in which survey questions were answered may have encouraged respondents to think of an average patient with less extreme characteristics.

We know that coding in medical records differs from physicians' notes on specific patients (9, 42, 43). Case identification based on ICD codes could overlook relevant cases and screening of clinical notes could improve knowledge of the patients' inflictions (42, 43). Confirmed diagnoses are rare (44–46). GPs find ICD-10-codes less precise than their “everyday clinical language” [p. 829, (47)] and often scribble detailed notes on patients in their reference files instead of using ICD codes that are often considered necessary for reimbursement purposes only (9, 18, 47, 48). Our study adds some insights into why GPs may not use ICD codes in the way they are expected to. Other studies find GPs' characteristics such as age, experience, working in individual or group practice and area of residence to be relevant for the way physicians handle patients with mental disorders, need for sedatives and chronic pain (22–26, 28, 29, 49). In contrast, we could not find any of these variables, apart from perceived exposure, knowledge of guidelines and experiences as a GP, to influence GPs subjective accounts of their handling of coding MUPS and somatoform disorders. This is somewhat reassuring, as it could indicate that German GPs offer a relatively homogeneous approach to the patients concerned. On the patient side, patients' preferences for terms describing symptoms differ (50) and also their tendency to be offended (51). Although further research is needed, this could also be relevant on GPs' side.

GPs' cautious coding behaviour (represented by items C3–C6) subjectively prevents them from, e.g., jeopardising patients' careers as public servants or the chances of getting a life insurance as medical records are checked for these purposes in Germany. Protecting patients from social disadvantages by avoiding specific ICD-codes is an aspect of GPs' daily practise also reported in the literature on patients with MUPS and somatoform disorders (9, 51–53). This behaviour appears to be mainly independent of sociodemographic variables and subjective exposure to patients with somatoform disorders. Its manifestation might be more influenced by the GPs' attitude towards MUPS and somatoform disorders or their (subjective) competency in diagnosing such disorders (9, 54). GPs seem to keep potential external readers of the patient records, e.g. insurance companies, in mind. In countries like Sweden, Spain, Estonia, Denmark, or the USA (e.g., in the Open Notes project- https://www.opennotes.org) the patient himself is a potential reader of his clinical records and although overall evaluation of this approach is positive (55), there are also some hints to physicians being more careful and less direct in their notes when they are aware of the patient as a potential consumer (55, 56). This might also become relevant in Germany with the introduction of the electronic health card in 2021 (57). Further research is needed to examine potential effects on GPs' coding behaviour concerning MUPS and somatoform disorders.

Not surprisingly, familiarity with diagnostic criteria and the subjective relevance of the diagnoses of somatoform disorders (represented by item C1 and C2) were influenced by knowledge of guidelines and the subjectively estimated number of patients with somatoform disorders in the GP's practice. Anyway, it is almost impossible to determine where the positive cycle of (subjective) high exposure to this kind of patients, awareness for the challenges of (not) diagnosing this patients, recognition of MUPS and somatoform disorders, and (guideline) knowledge begins. A high proportion of patients with MUPS or somatoform disorder can lead to increased engagement with and thereby better knowledge of the topic, while increased engagement itself could lead to a higher awareness and better recognition of the affected patients.

Other European countries (e.g., France or Denmark) do not use ICD-10-coding in primary care, but classify reasons for encounters, diagnosis/problems managed and performed intervention using the International Classification pf Primary Care [ICPC, (58)]. This might lead to a very different approach in handling notes on and coding of MUPS/somatoform disorders and maybe even diagnosing somatoform disorders in these countries. Also, different countries have different approaches and guidelines in use. The Dutch guideline on medically unexplained symptoms (59) considers medically unexplained symptoms to be a working hypotheses based on the assumed exclusion of somatic/psychiatric pathology, to be reconsidered when symptoms change or become critical. The ICD-10-coding (30) used in Germany also allows for tentative diagnoses, which reflect the Dutch approach to using MUPS as a working hypothesis (59).

### Implications for Practice

Considering that knowledge of guidelines positively influences the coding behaviour of GPs in our study, facilitating the knowledge and implementation of guidelines (39) through training and organisational changes in the primary care team (60) can be seen as a helpful measure. We know that the dissemination and implementation of guidelines remain difficult (61, 62), especially in primary care, where so many different and all too often specialised guidelines need to be considered.

In contrast to the assumption that more adequate coding leads to better health care (16) not all German GPs view ICD-10-coding as a prerequisite for treating patients with MUPS and somatoform disorders (9). Our study shows, that handling and coding of MUPS and somatoform disorders might change with GPs' growing experience. GPs' experience with and subjective exposure to patients with MUPS or somatoform disorders can hardly be externally influenced, but positive effects could be imagined, for example, through simulated patient encounters. A challenge to this approach is that somatoform disorders may be difficult to simulate (63). In any case, optimism should be cautious, since there are some hints that training GPs might improve attitudes towards patients with MUPS (4, 64), but many training programs do not show positive effects on clinical outcomes (65–67) and patient satisfaction (65). Either way, our results and the results of many other studies in the field (4, 5, 68) point to the need of further educating GPs on coding, handling and diagnosing MUPS and somatoform disorders and (with the upcoming changes in the ICD-11 soon to be implemented in Germany) bodily distress disorders.

## Data Availability Statement

The dataset presented in this article is not readily available because the data that support the findings of this study are not yet publicly available. Requests to access the dataset should be directed to Nadine J. Pohontsch (n.pohontsch@uke.de).

## Ethics Statement

The studies involving human participants were reviewed and approved by Ethics Committee of the Hamburg Medical Association. Participation was completely voluntary and anonymous. Participants were notified before participation that their informed consent would be assumed if they voluntarily returned a completed response form.

## Author Contributions

BL and MS: acquisition of funding for and conception, design and supervision of the study. KK, ML, NP, LR, and TZ: acquisition of data. NP and TZ: data analysis and interpretation, drafting of the manuscript. KK, BL, ML, LR, and MS: critical revision of former versions and final approval of the manuscript. All authors contributed to the article and approved the submitted version.

## Conflict of Interest

The authors declare that the research was conducted in the absence of any commercial or financial relationships that could be construed as a potential conflict of interest.
