# Supplementary material for: ICD-10-Coding of Medically Unexplained Physical Symptoms and Somatoform Disorders—A Survey With German GPs
Source: Front Med (Lausanne). 2021 Mar 30;8:598810. doi: 10.3389/fmed.2021.598810 (PMC8042316; doi:10.3389/fmed.2021.598810)
Supplement: Supplementary file 1 [file Data_Sheet_1.PDF]

Supplementary Figure 1: Descriptive statistics of coding Items

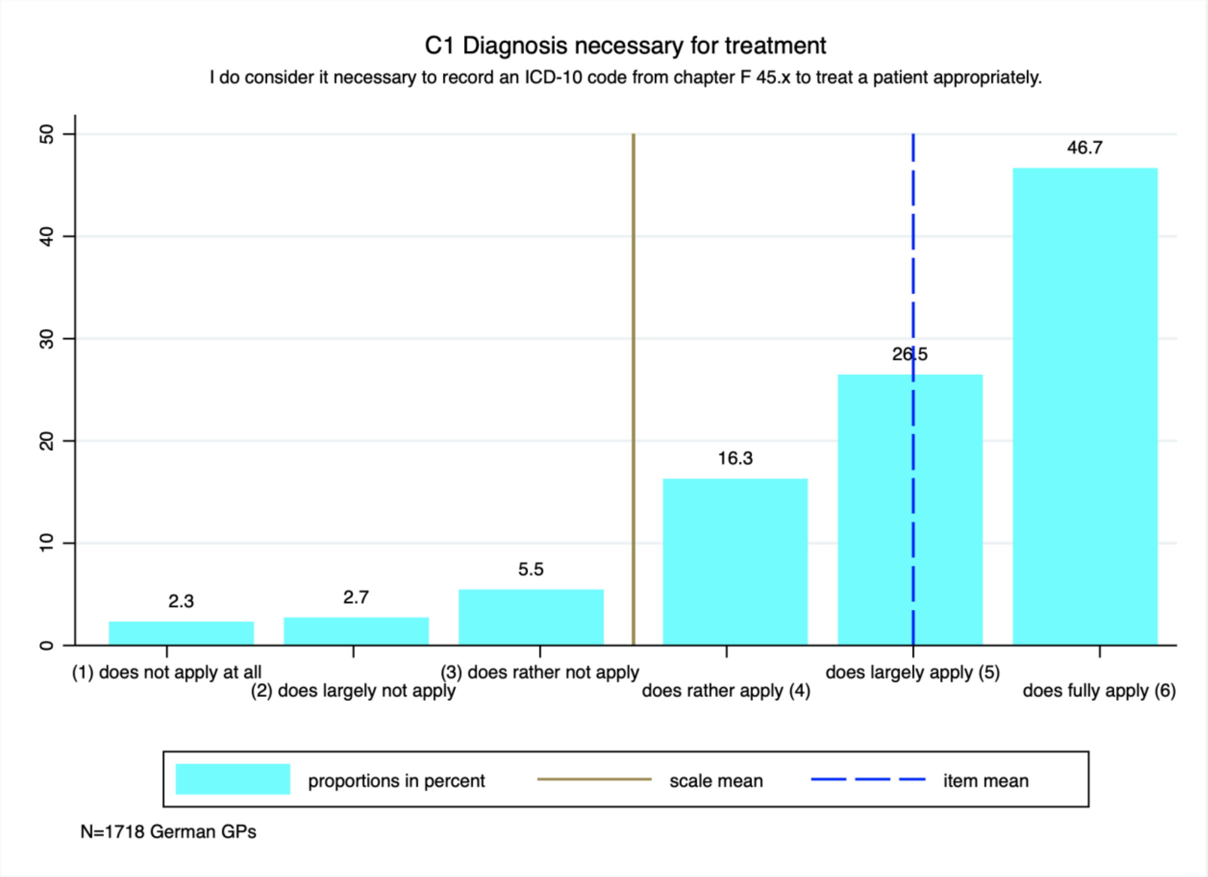

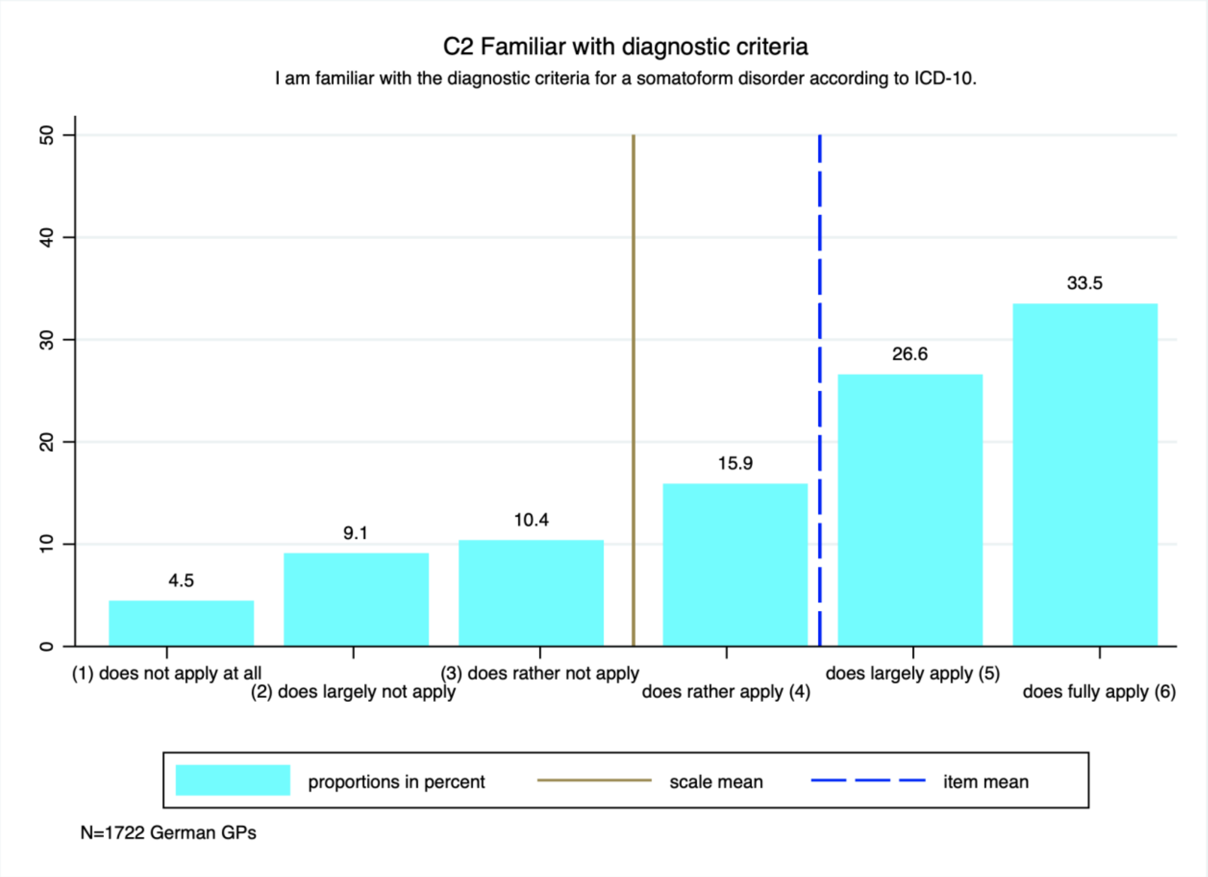

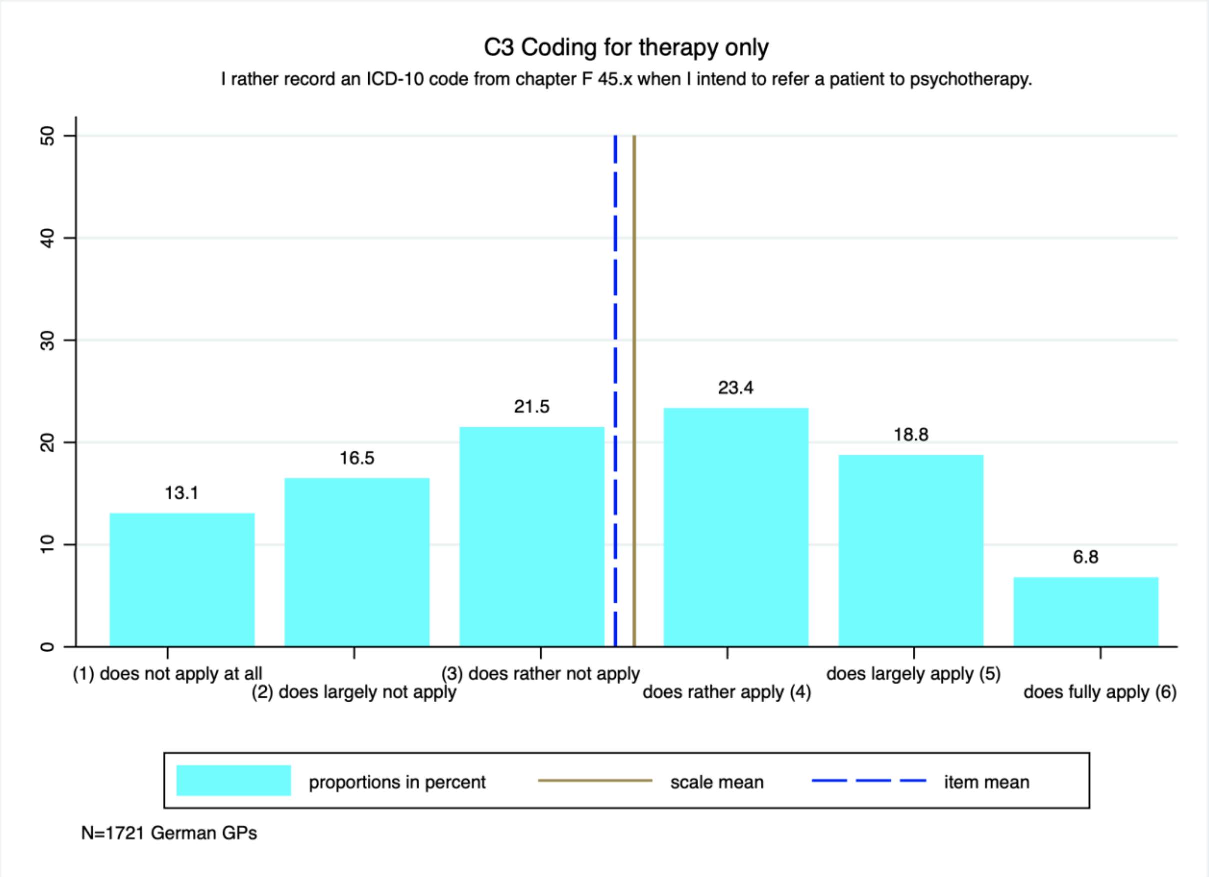
C3 Coding for therapy only
I rather record an ICD-10 code from chapter F 45.x when I intend to refer a patient to psychotherapy.
0
10
20
30
40
50
13.1
16.5
21.5
23.4
18.8
6.8
(1) does not apply at all
(2) does largely not apply
(3) does rather not apply
does rather apply (4)
does largely apply (5)
does fully apply (6)
proportions in percent
scale mean
item mean
N=1721 German GPs

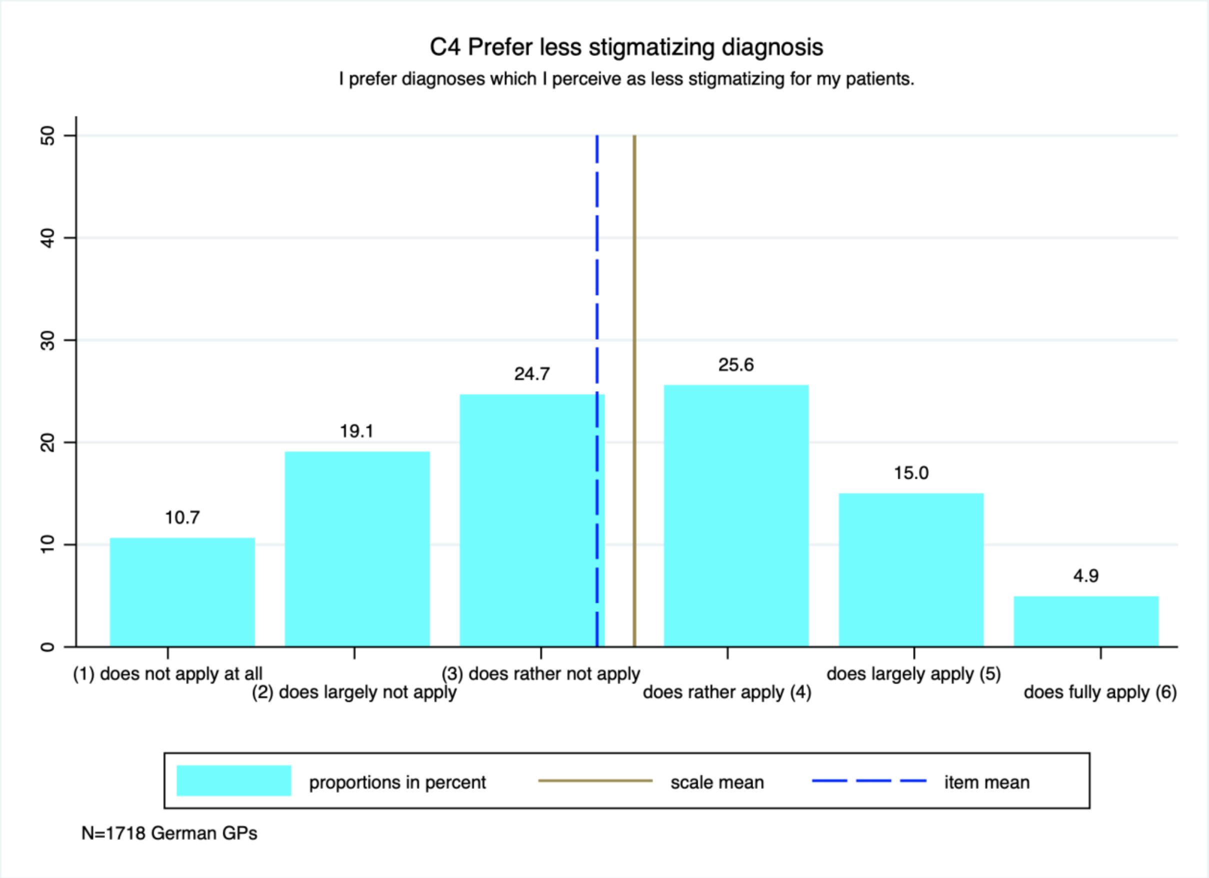
C4 Prefer less stigmatizing diagnosis
I prefer diagnoses which I perceive as less stigmatizing for my patients.
0
10
20
30
40
50
10.7
19.1
24.7
25.6
15.0
4.9
(1) does not apply at all
(2) does largely not apply
(3) does rather not apply
does rather apply (4)
does largely apply (5)
does fully apply (6)
proportions in percent
scale mean
item mean
N=1718 German GPs

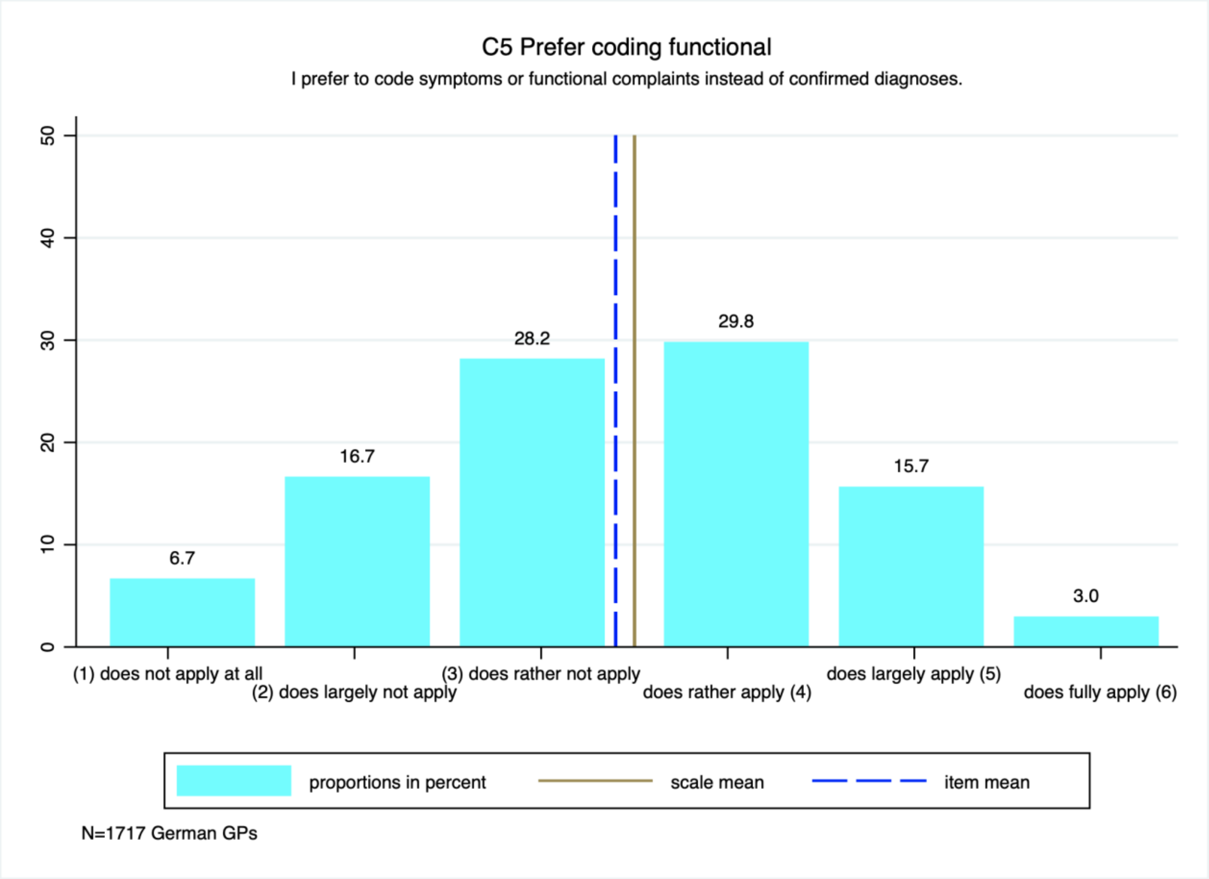
C5 Prefer coding functional
I prefer to code symptoms or functional complaints instead of confirmed diagnoses.
0
10
20
30
40
50
6.7
16.7
28.2
29.8
15.7
3.0
(1) does not apply at all
(2) does largely not apply
(3) does rather not apply
does rather apply (4)
does largely apply (5)
does fully apply (6)
proportions in percent
scale mean
item mean
N=1717 German GPs

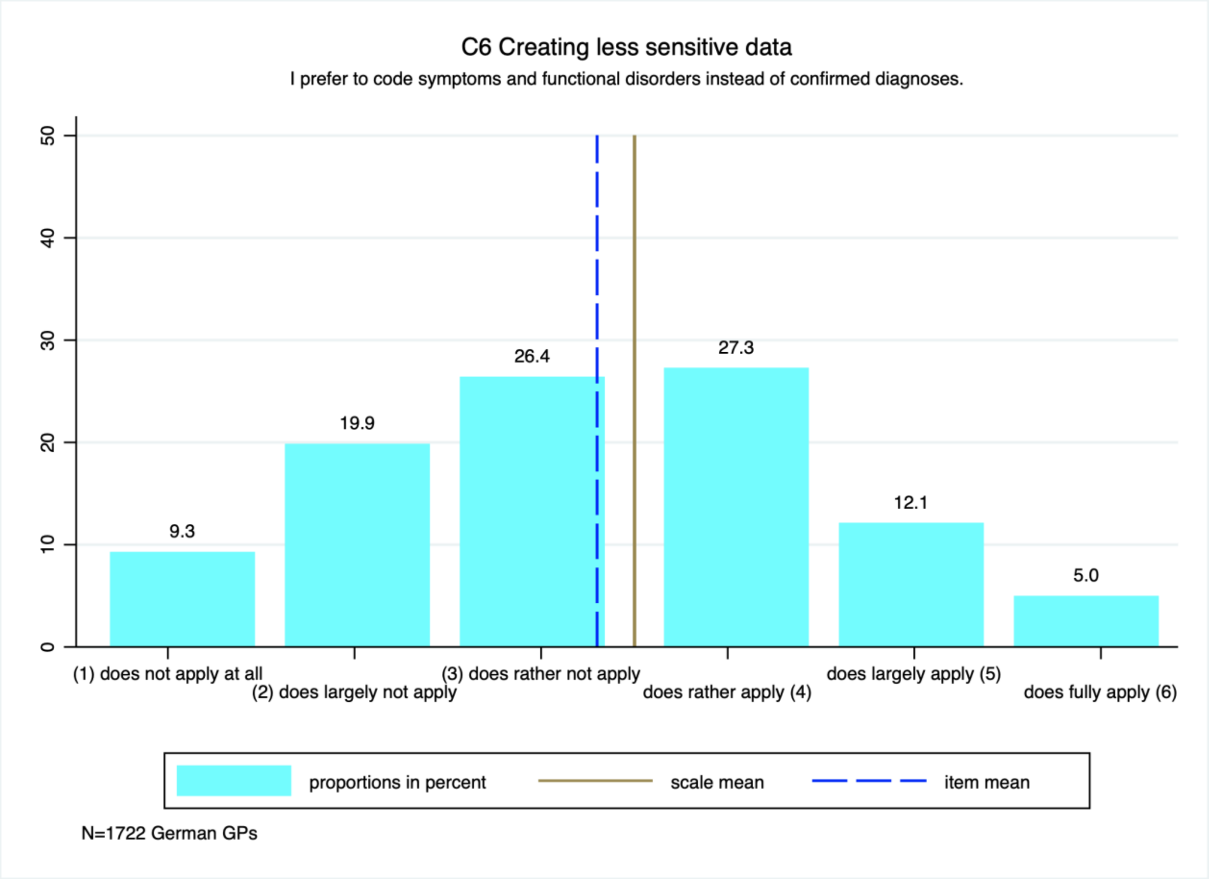
C6 Creating less sensitive data
I prefer to code symptoms and functional disorders instead of confirmed diagnoses.
0
10
20
30
40
50
9.3
19.9
26.4
27.3
12.1
5.0
(1) does not apply at all
(2) does largely not apply
(3) does rather not apply
does rather apply (4)
does largely apply (5)
does fully apply (6)
proportions in percent
scale mean
item mean
N=1722 German GPs
