# Supplementary material for: ICD-10-Coding of Medically Unexplained Physical Symptoms and Somatoform Disorders—A Survey With German GPs
Source: Front Med (Lausanne). 2021 Mar 30;8:598810. doi: 10.3389/fmed.2021.598810 (PMC8042316; doi:10.3389/fmed.2021.598810)
Supplement: Supplementary file 2 [file Data_Sheet_2.PDF]

Supplementary Table 1: Results for the logistic regression of coding items (odds ratios)

	Odds ratio	Std. Err.	p-value	95%-CI-below	95%-CI-above
**C1) I do not consider recording a ICD-10 code from chapter F 45.- (somatoform disorders) necessary to treat a patient with a somatoform disorder adequately** *(results inverted)*. **(N=1038)**					
Sex = female	1.91	0.82	0.133	0.82	4.42
Experience as GP in years	1.01	0.01	0.565	0.98	1.04
Interaction experience * sex	0.99	0.02	0.757	0.95	1.04
Certified GP = yes	1.06	0.24	0.799	0.68	1.66
Knowledge of guidelines	1.03	0.06	0.612	0.92	1.16
Basic psychosomatic care = yes	0.75	0.26	0.399	0.38	1.46
Practice setting (other than single)	0.92	0.20	0.709	0.61	1.40
Proportion of patients with somatoform disorders ≥10%	1.72	0.38	0.013*	1.12	2.64
**C2) I am not familiar with the diagnostic criteria for a somatoform disorder according to the ICD-10** *(results inverted)*. **(N=1042)**					
Sex = female	1.06	0.32	0.843	0.58	1.93
Experience as GP in years	1.01	0.01	0.405	0.99	1.03
Interaction experience * sex	1.00	0.01	0.947	0.97	1.03
Certified GP = yes	1.09	0.18	0.586	0.80	1.50
Knowledge of guidelines	1.18	0.05	0.001**	1.09	1.29
Basic psychosomatic care = yes	1.16	0.25	0.491	0.76	1.79
Practice setting (other than single)	0.91	0.14	0.529	0.68	1.22
Proportion of patients with somatoform disorders ≥10%	1.41	0.21	0.022*	1.05	1.90

**C3) I rather record an ICD-10 code from chapter F 45.- (somatoform disorders) when I intend to refer a patient to psychotherapy. (N=1038)**					
Sex = female	0.67	0.17	0.119	0.40	1.11
Experience as GP in years	1.00	0.01	0.603	0.98	1.01
Interaction experience * sex	1.01	0.01	0.256	0.99	1.04
Certified GP = yes	1.02	0.14	0.886	0.78	1.33
Knowledge of guidelines	1.06	0.04	0.089	0.99	1.13
Basic psychosomatic care = yes	0.91	0.17	0.628	0.63	1.32
Practice setting (other than single)	1.17	0.15	0.204	0.92	1.50
Proportion of patients with somatoform disorders ≥10%	0.86	0.11	0.242	0.67	1.10
**C4) I prefer diagnoses which I perceive as less stigmatizing for my patients. (N=1036)**					
Sex = female	1.50	0.40	0.126	0.89	2.52
Experience as GP in years	1.02	0.01	0.049*	1.00	1.04
Interaction experience * sex	1.00	0.01	0.785	0.97	1.02
Certified GP = yes	0.87	0.12	0.301	0.66	1.14
Knowledge of guidelines	0.99	0.03	0.817	0.93	1.06
Basic psychosomatic care = yes	1.16	0.22	0.454	0.79	1.69
Practice setting (other than single)	1.07	0.14	0.588	0.84	1.37
Proportion of patients with somatoform disorders ≥10%	0.81	0.10	0.100	0.63	1.04

**C5) For patients with persistent, non-specific and bothersome somatic symptoms I prefer to code symptoms and functional disorders instead of confirmed diagnoses. (N=1038)**					
Sex = female	1.37	0.36	0.223	0.82	2.29
Experience as GP in years	1.00	0.01	0.797	0.98	1.02
Interaction experience * sex	0.98	0.01	0.061	0.95	1.00
Certified GP = yes	1.20	0.16	0.187	0.92	1.57
Knowledge of guidelines	0.93	0.03	0.034*	0.87	0.99
Basic psychosomatic care = yes	1.30	0.25	0.177	0.89	1.89
Practice setting (other than single)	1.14	0.14	0.299	0.89	1.46
Proportion of patients with somatoform disorders ≥10%	0.89	0.11	0.361	0.70	1.14
**C6) I prefer diagnoses which I consider as less compromising for the patients' further life course (for example, insurances, civil service career). (N=1042)**					
Sex = female	0.94	0.24	0.816	0.57	1.56
Experience as GP in years	1.00	0.01	0.668	0.99	1.02
Interaction experience * sex	1.00	0.01	0.866	0.97	1.02
Certified GP = yes	0.82	0.11	0.132	0.62	1.06
Knowledge of guidelines	1.01	0.03	0.743	0.95	1.08
Basic psychosomatic care = yes	1.38	0.27	0.092	0.95	2.02
Practice setting (other than single)	1.14	0.14	0.300	0.89	1.46
Proportion of patients with somatoform disorders ≥10%	0.96	0.12	0.715	0.75	1.22

* significance level <0.05; ** significance level <0.001
